# Crater detection from commercial satellite imagery to estimate unexploded ordnance in Cambodian agricultural land

**DOI:** 10.1371/journal.pone.0229826

**Published:** 2020-03-18

**Authors:** Erin Lin, Rongjun Qin, Jared Edgerton, Deren Kong

**Affiliations:** 1 Department of Political Science, The Ohio State University, Columbus, Ohio, United States of America; 2 Department of Civil, Environmental and Geodetic Engineering & Department of Electrical and Computer Engineering, The Ohio State University, Columbus, Ohio, United States of America; Beijing University of Technology, CHINA

## Abstract

Unexploded ordnance (UXO) pose a significant threat to post-conflict communities, and current efforts to locate bombs rely on time-intensive and dangerous in-person enumeration. Very high resolution (VHR) sub-meter satellite images may offer a low-cost and high-efficiency approach to automatically detect craters and estimate UXO density. Machine-learning methods from the meteor crater literature are ill-suited to find bomb craters, which are smaller than meteor craters and have high appearance variation, particularly in spectral reflectance and shape, due to the complex terrain environment. A two-stage learning-based framework is created to address these challenges. First, a simple and loose statistical classifier based on histogram of oriented gradient (HOG) and spectral information is used for a first pass of crater recognition. In a second stage, a patch-dependent novel spatial feature is developed through dynamic mean-shift segmentation and SIFT descriptors. We apply the model to a multispectral WorldView-2 image of a Cambodian village, which was heavily bombed during the Vietnam War. The proposed method increased true bomb crater detection by over 160 percent. Comparative analysis demonstrates that our method significantly outperforms typical object-recognition algorithms and can be used for wide-area bomb crater detection. Our model, combined with declassified records and demining reports, suggests that 44 to 50 percent of the bombs in the vicinity of this particular Cambodian village may remain unexploded.

## Introduction

Unexploded ordnance (UXO) are defined as military explosives, such as grenades, bombs, mortar shells and cluster munitions, that are deployed during armed conflict but fail to detonate, and UXO pose significant challenges to post-war economic recovery, human health and welfare, and government responsiveness. Each year, UXO claim the lives of 15,000 to 20,000 people, and the majority of victims are children or civilians [1]. The presence of UXO in agricultural fields extends the cost of war to long-term crop production, as field inaccessibility reduces agricultural production by millions of US dollars in the Middle East [2]. Practitioners have observed that the latent risk of bomb explosion makes it dangerous for government providers to respond to local demands for services [3].

It has been suggested that Cambodia has some of the highest contamination rates in the world. The United States dropped an estimated 500,000 tons of explosives on Cambodia during the Vietnam War. All 24 provinces still have areas contaminated with unexploded ordnance and mines, and in 2001, almost half of all Cambodian villages reported some form of UXO-contamination [4]. Current land clearance methods use laborious and often inefficient means to find contaminated, high-density areas. Removal practices require deminers to manually search fields, relying on metal and radar detectors to find possible bombs and using shovels to carefully dig out the suspected explosives [5]. A 2016 United Nations report found that nearly half of the area cleared in the past year “contained no or a very limited number of mines” [6]. As a result, an estimated four to six million stray explosives have not yet been located. An average of more than two civilians are killed or injured by UXO each day, and 28 percent of the casualties are children [4].

Remote-sensing analysis provides an alternative means to locate UXO. Declassified US Air Force records of Vietnam War bombing runs have been used to estimate the effectiveness of airstrikes on insurgent attacks, civilian political attitudes, and capital recovery [7–9]. However the records’ coordinates of the payload drops have not been applied to the literature on UXO identification [10, 11], which develops field equipment to magnetically sense UXO but does not provide an *ex ante* measure of high-density areas. To address this challenge, this article develops a remote-sensing method to count the number of bomb craters (a proxy for detonated bombs) in each payload’s target zone. Once the number of detonated bombs is subtracted from the total bombs in each payload (information provided by the declassified data), the number of bombs still unaccounted for and potentially hidden in the drop zone can be estimated.

Previous attempts to detect bomb craters borrow from well-established methods in the meteor crater literature, which scan satellite images for large, circular craters on planetary surfaces in outer space [12–15]. Key differences between bomb craters and meteor craters may result in these methods undercounting the bomb craters on satellite images. First, bomb craters experience various levels of erosion and vegetal overgrowth over time, unlike meteor craters, which are situated on extraterrestrial surfaces that lack atmosphere and vegetation. In other words, bomb craters have high appearance variation, or intra-class variation. Second, bomb craters are relatively small in size from a remote sensing perspective, typically only 3 to 12 meters in diameter [16, 17] and much harder to find than meteor impact craters, which can be up to 3,000 meters in diameter.

**Fig 1:**
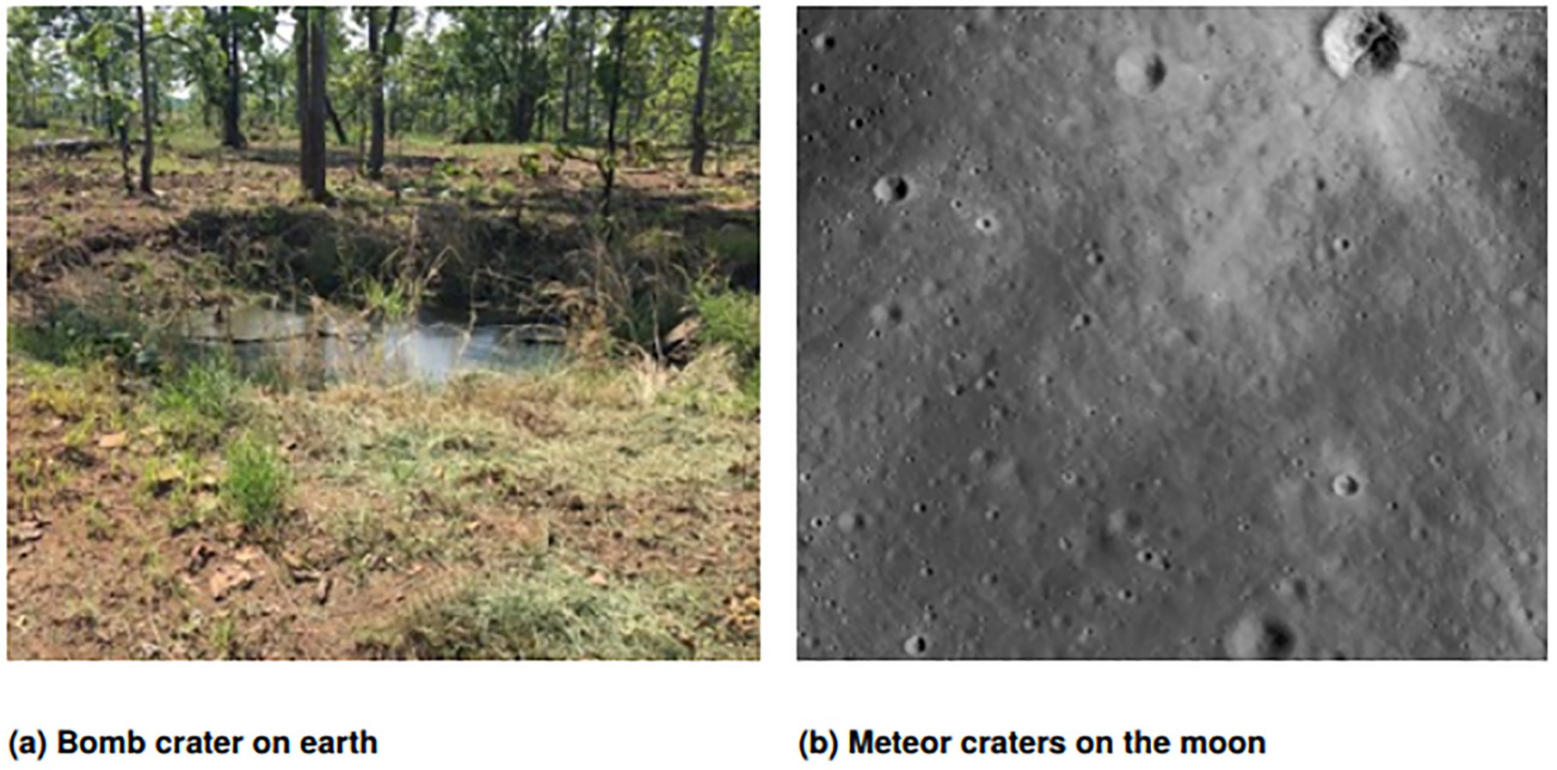
Photographs of (a) a bomb crater in Cambodia [author’s image] and (b) meteor craters on moon [from NASA’s Earth Observatory Database [21]]. Meteor craters tend to be more precisely circular and do not experience erosion, suggesting that bomb craters require an alternative method of detection.

Since meteor crater methods detect circular shapes from coarse-grained, black-and-white images, this purely heuristic approach likely misses bomb craters that are smaller in size, that do not have a perfectly circular shape, that blend into the surrounding terrain, or that have disturbance objects (e.g., plants or water) in or near the crater. Fig 1 provides examples that illustrate these differences between bomb craters and meteor craters. Higher resolution and geometric data (i.e., LiDAR) can demarcate conflict areas with some success [18, 19]. But in order to detect an object as small as 3 to 12 meters in diameter, researchers need to work with Very High Resolution (VHR) satellite images, as craters on VHR images are roughly the equivalent of 100 pixels in size, which provides enough information to detect a variety of feature patterns with remote-sensing methods. Like in recent scholarship, Very High Resolution images are defined as remote sensing data with a spatial resolution of 0.3 to 1 meter [20].

A machine-learning based detection framework draws on the advantages of VHR images by detecting bomb craters through building classifiers based on specially designed features—a particularly well-suited method, given that crater detection is a target-specific learning task with a relatively small number of samples available. Since bomb craters generally follow isotropic patterns, the framework considers both shapes and appearances features, including circular shapes [22–24], contours [25], morphological features [26], and gradients [27]. When building these custom features, this framework accommodates the variation of shapes and surrounding objects since some craters have eroded or have been planted in the fifty years following the bombing. But by including a wider variation in shapes and appearances features in the classifier, the pool of crater candidates is also expected to contain more false positives. The classifier must be able to include the many types of true positives (that were likely missed in purely heuristic models) while also filtering out the false positives (that result from the more inclusive selection mechanism).

This article provides an innovative model structure built to achieve these objectives. Since standard statistical learning models cannot typically accommodate the data variation presented in bomb craters—due to the fact that single stage learning models do not allow for subsequent refinement on the feature level—an alternative framework is created. Recent research shows that hierarchical learning models, such as decision trees and random forests, outperform statistical classifiers when dealing with multi-modal features (like appearance and geometry) and non-continuous features [28, 29].

Therefore, a two-stage framework is developed for our learning method. In the first stage, a first pass of bomb crater candidates is extracted from the 100 square-kilometer study area by creating patches with a sliding window technique, in which a rectangular region slides across an image with a fixed width and height. The patches are then classified into either potential craters or rejected candidates. Specifically, a typical feature extractor concatenates a histogram of oriented gradient (HOG) with a spectrum histogram feature vector for support vector machine (SVM) based classification, which has reported better accuracy with spectrum value based land-cover classification when compared to alternative methods [30].

As mentioned earlier, the potential crater candidates likely contains many false positives. Thus, the second stage involves a multi-method process to remove the non-craters from the candidate pool. First, a simple SVM classifier eliminates easily recognizable false positives, such as buildings and trees. Then, a novel feature descriptor is crafted specifically for crater shape pattern identification. It is assumed that craters are approximately circular in shape with very small singular regions that may have different textures, caused by variation in shade, water, and terrain [17]. Building on feature space analysis [31], a robust adaptive mean-shift-based shape (AMSBS) feature is developed to separate the different regions in a crater candidate. Then a location-specific Scale Invariant Feature Transform (SIFT) feature descriptor is applied to best describe the texture of the regions, and concatenate it to the AMSBS feature vector. Finally, a binary classification is performed on the final pool of crater candidates, using a sum-of-trees model, specifically random forest, which is more suitable for multi-modal data.

The rest of the paper is organized as follows. Section 2 introduces the experimental dataset, including the training and validation data collected from the satellite image. Section 3 presents the proposed two-stage framework, which includes the novel patch dependent AMSBS feature. Section 4 provides the experimental results. Section 5 uses the crater results in a real-world application, estimating the number of UXO left in the study site. When our results are paired with land classification data, we find that the majority of the contaminated land is actively cultivated, suggesting that demining services should target this high-use area. Finally, the article discusses the benefits and scope conditions of our proposed method as well as applications to other real-world problems, and concludes.

## Study site and experimental data

**Fig 2:**
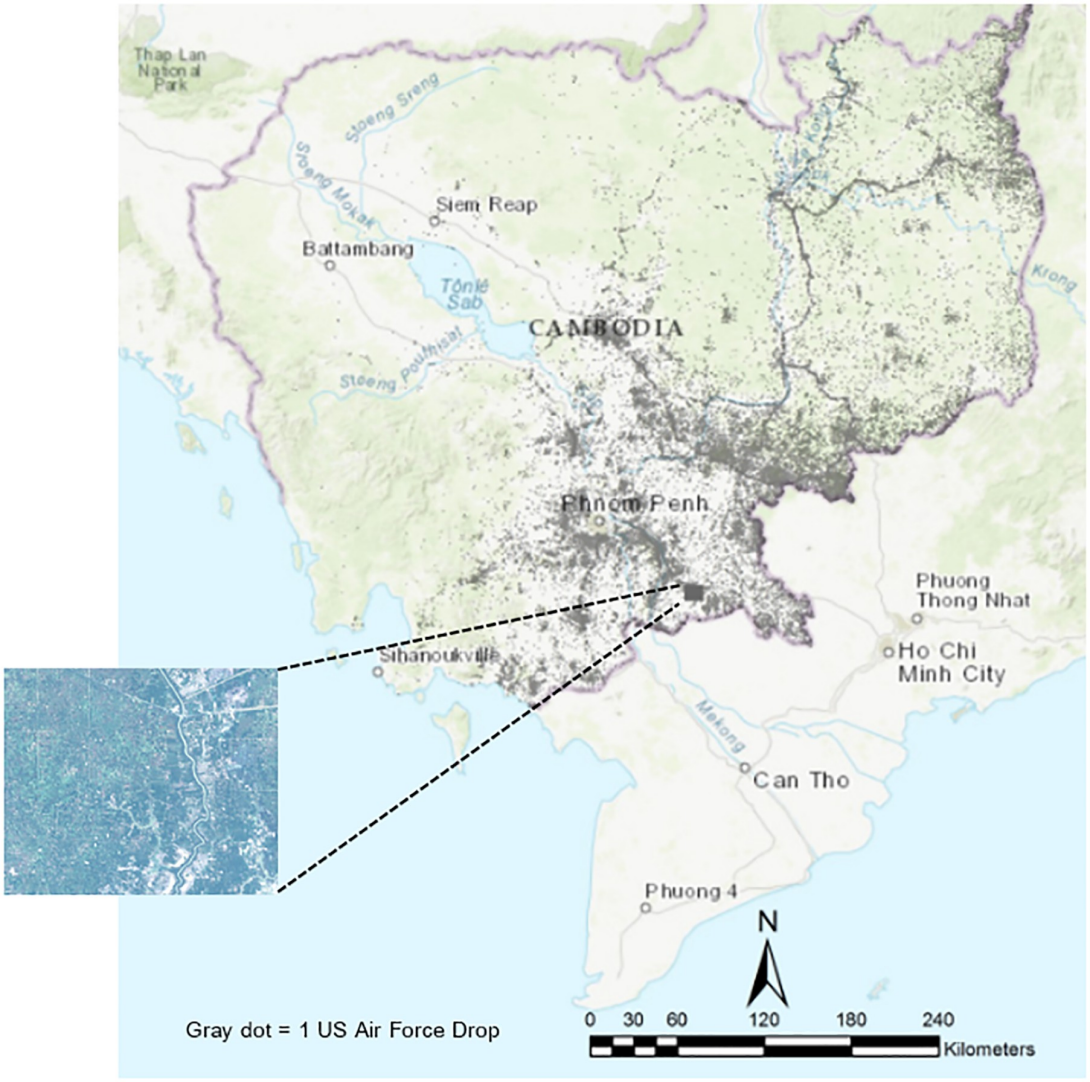
The Very High Resolution (VHR) satellite image is located in Prey Veng province, Cambodia, part of a heavily bombed area roughly 30 kilometers from the Vietnam border. Each gray dot represents one of 113,716 payloads dropped over Cambodia from 1965 to 1973. Basemap from USGS National Boundaries Dataset (URL: https://viewer.nationalmap.gov/advanced-viewer/).

To build this two-stage framework, the article draws on experimental data from a WorldView-2 multispectral image of Kampong Trabaek town in Prey Veng province, Cambodia. This VHR image covers an area of 100 km^2^ (0.5 meter ground sampling distance). The date of data acquisition was July 4, 2015, and its radiometric resolution is 16-bit. The bands and their wavelength used for this study are near-infrared (770-895 nm), R (630-690 nm), G (510-589 nm), and B (450-510 nm). Geometric and radiometric correction is performed at level 1. As shown in Fig 2, the image is located in southeastern Cambodia, roughly 30 kilometers away from the Vietnam border, and is one example of the many areas in the eastern half of the country that experienced heavy bombing.

The declassified US Air Force records reveal that 3,205 general purpose bombs (more commonly known as carpet bombs) were dropped within this 100 km^2^ area. The bombing was part of the US 7th Air Force interdiction and close air support campaign from May 1970 to August 1973, also known as Operation Freedom Deal. Although the campaign was initially restricted to within 50 kilometers of the South Vietnam border, after two months the operation moved west past the Mekong River and covered the majority of the country—all in an effort to sever the People’s Army of Vietnam supply lines that ran through Cambodia and Laos.

**Fig 3:**
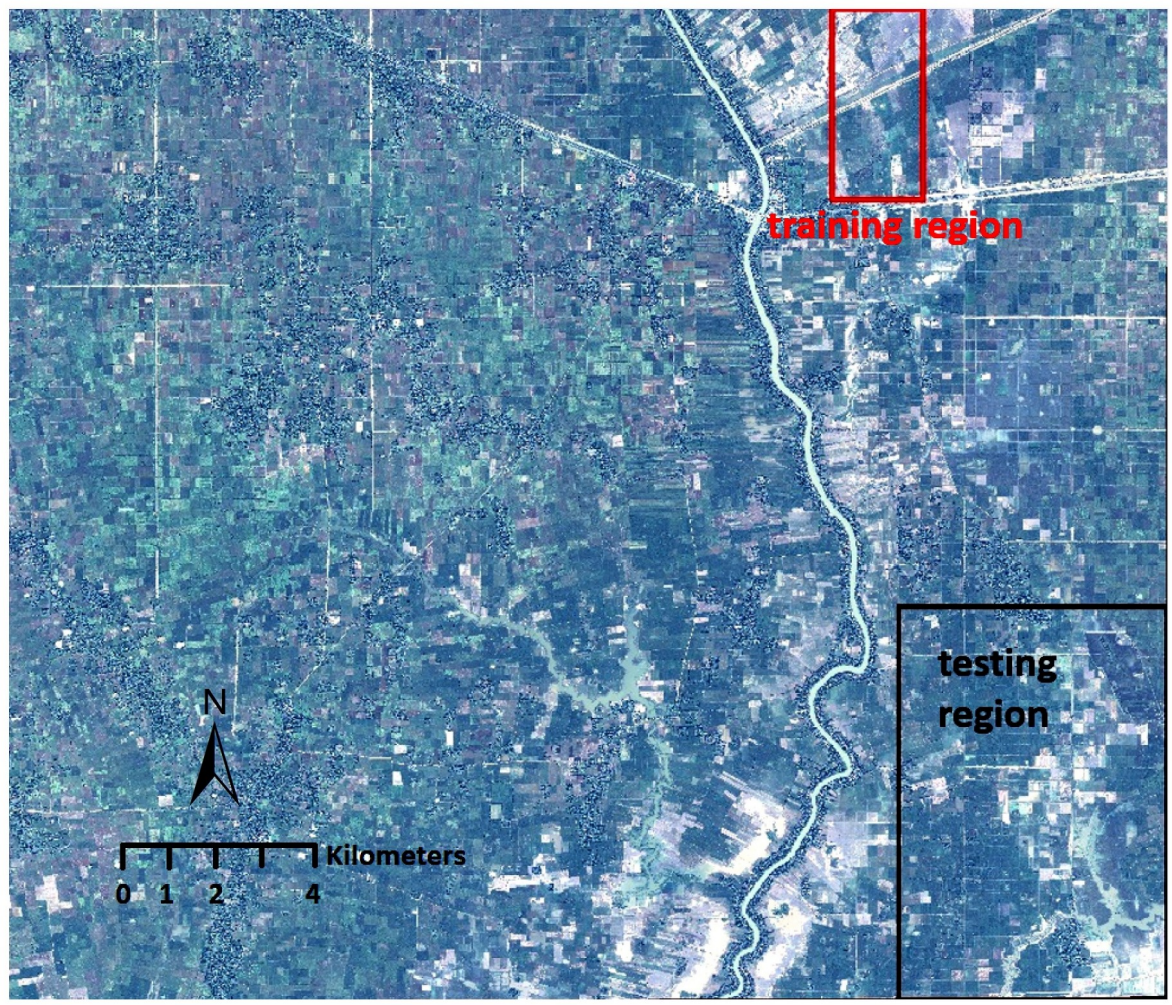
The satellite image (100 km^2^) of the study site. After we built and evaluated the two-stage model on the training and validation region, detection was performed over the entire region.

There are, of course, limits to any single study site. Yet there are two reasons to believe that the Prey Veng location and the model built from data generated from this site have external validity. First, the site provides an array of terrains—most notably rice paddies, peri-urban development, and river floodplains—that surround existing bomb craters. Fig 3 provides a closer look at the entire satellite image. The Kampong Trabaek river runs north to south, irrigating the region’s rice paddies. Kampong Trabaek town (population 1,358) lies due south of the training region, at the intersection of Route 1 and the river that shares its name. The wood and metal buildings, water features, and trees are common disturbances that will be incorporated into the model.

Second, this area represents a “most likely” case for finding a high ratio of undetonated to detonated bombs. Only 119,857 m^2^ (or 0.12% of the image) have been cleared by professional deminers, despite the Cambodian Mine Action Center labelling this region as a high priority. Within that cleared space, deminers found two general purpose bombs and hundreds of scrap metal pieces. This ratio highlights the recurring inefficiency in the clearance process, particularly the difficulty that deminers face in distinguishing bombs from leftover metals. Despite the substantive importance of aiding the demining process in Kampong Trabaek and its surrounding fields, the proposed method may also be applicable to a wide range of cases, a claim that nonetheless requires further comparative study.

We selected two regions from the satellite image in which to collect training and validation data. These regions were chosen according to their proximity to the river (flooded craters tend to be closer to the water source), intersection with a road (which provides more buildings and urban disturbances), and mix of active and inactive rice paddies (leading to color variation of green and brown craters). Fig 3 shows the regions where the model was trained and validated, before it ran on the entire satellite image. The size of the training and validation region are approximately 1757 × 3554 and 5206 × 7394 pixels of the entire satellite image (ca. 22666 × 18524 pixels), making the training and validation sets statistically significant and representative of the overall image.

**Fig 4:**
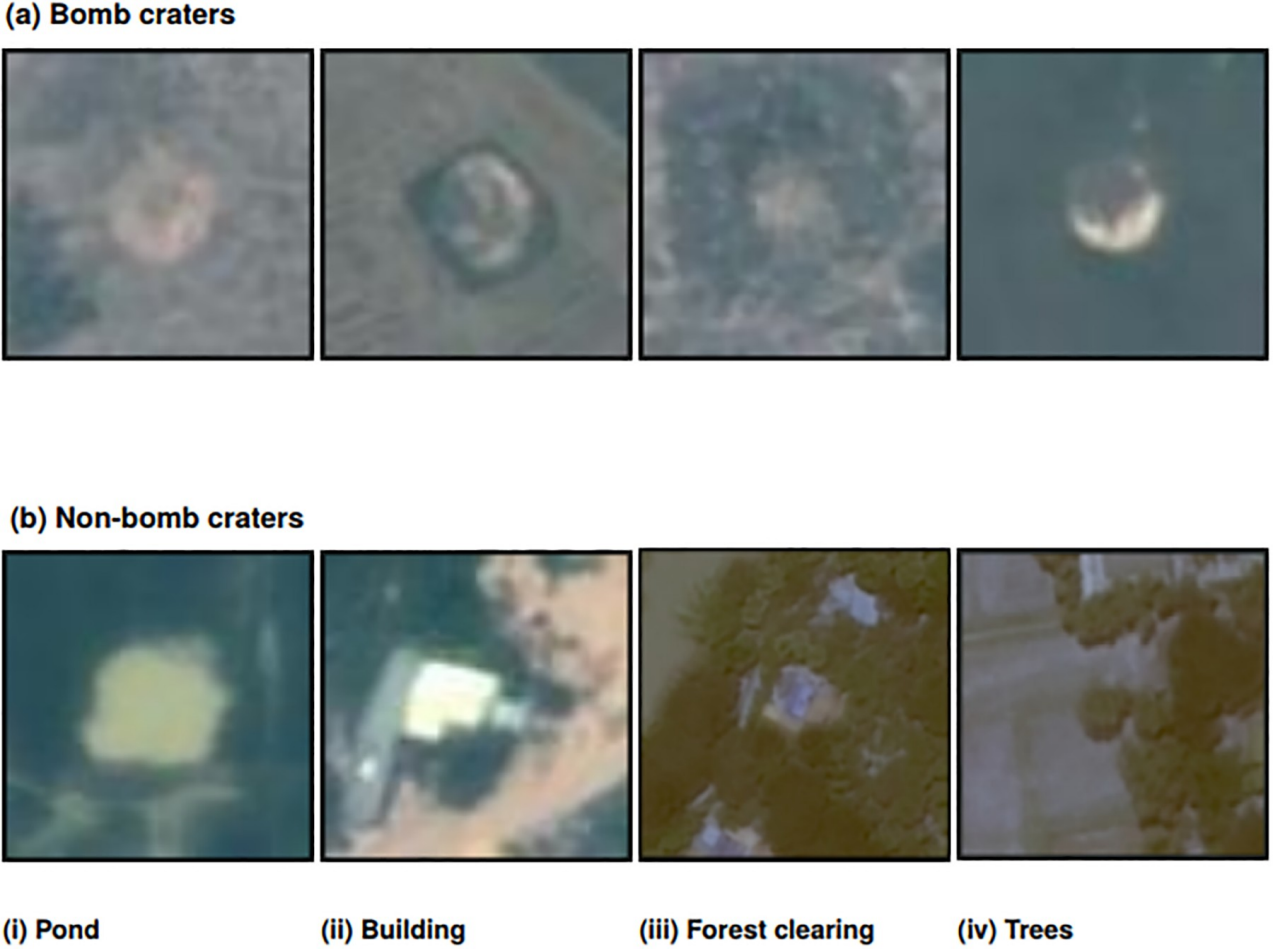
Example patches of correctly identified bomb craters (a) and falsely identified bomb craters (b). The selection of false bomb crater images include a building, pond, and trees from the first stage classification.

To create the training and validation datasets, craters were labeled with the best manual effort in small image patches of 64 × 64 pixels. This is equivalent to a 32 × 32 m^2^ footprint in the WorldView satellite image, which captures the size of the largest craters, approximately 12 meters in diameter. The human coder was provided an initial sample of ground-truthed crater images, verified through international demining organizations working in Cambodia, and used them to identify 49 positive crater images and 108 negative crater images in the training region. An image patch was coded as a positive sample if a bomb crater appears in its center; otherwise it was regarded as a negative sample. Fig 4 provides some examples of positive and negative crater images from the training data.

To expand the training data, we performed several data augmentations on the labeled crater image patches. This included horizontal flipping and rotating of the training data patches 90, 180, and 270 degrees. The data augmentations help to avoid patch rotation dependence and over-fitting [32]. This expanded the original training data from 157 to 1,256 samples. Then the model was run on the validation region, outlined in black in Fig 3 above. The algorithm’s output was checked against the human coder’s labels, and the model was further refined before it was run over the entire satellite image. The two-stage crater detection framework is described in detail in the next section. The model’s results and its performance statistics compared to alternative approaches are provided in the section after that.

## Methodology

The study is composed of two major methodological stages: (i) support vector machine (SVM) classification to identify patches with circular shapes of various colors, and (ii) a novel classification method that extracts texture, color, and location information from a variety of circular sizes within an image patch, using adaptive segmentation to detect circular objects, extracting central scale-invariant feature transform (SIFT) points and adaptive mean-shift-based shape features, and classifying with a random forest model.

In the first step, a sliding window extracts image patches from the satellite image. Then, a support vector machine (SVM) algorithm detects circular or near-circular objects with spectral values that match the sample craters. This method follows standard SVM classification, based on Histogram of Gradient (HOG) and spectral information. We expect the SVM classification to include several false positives because conventional classification methods typically extract feature vectors using all pixels in the patch, so other circular objects, like ponds and buildings, were detected in model building. Therefore, disturbances surrounding a bomb crater (e.g., trees or small buildings that lie in the corner of the patch) will be absorbed in the feature vector and bias the extracted data. By the end of the first stage, the model has sorted the patches into preliminary groups of true and false candidates, which we will use to compare our first-stage results with two alternative approaches.

**Fig 5:**
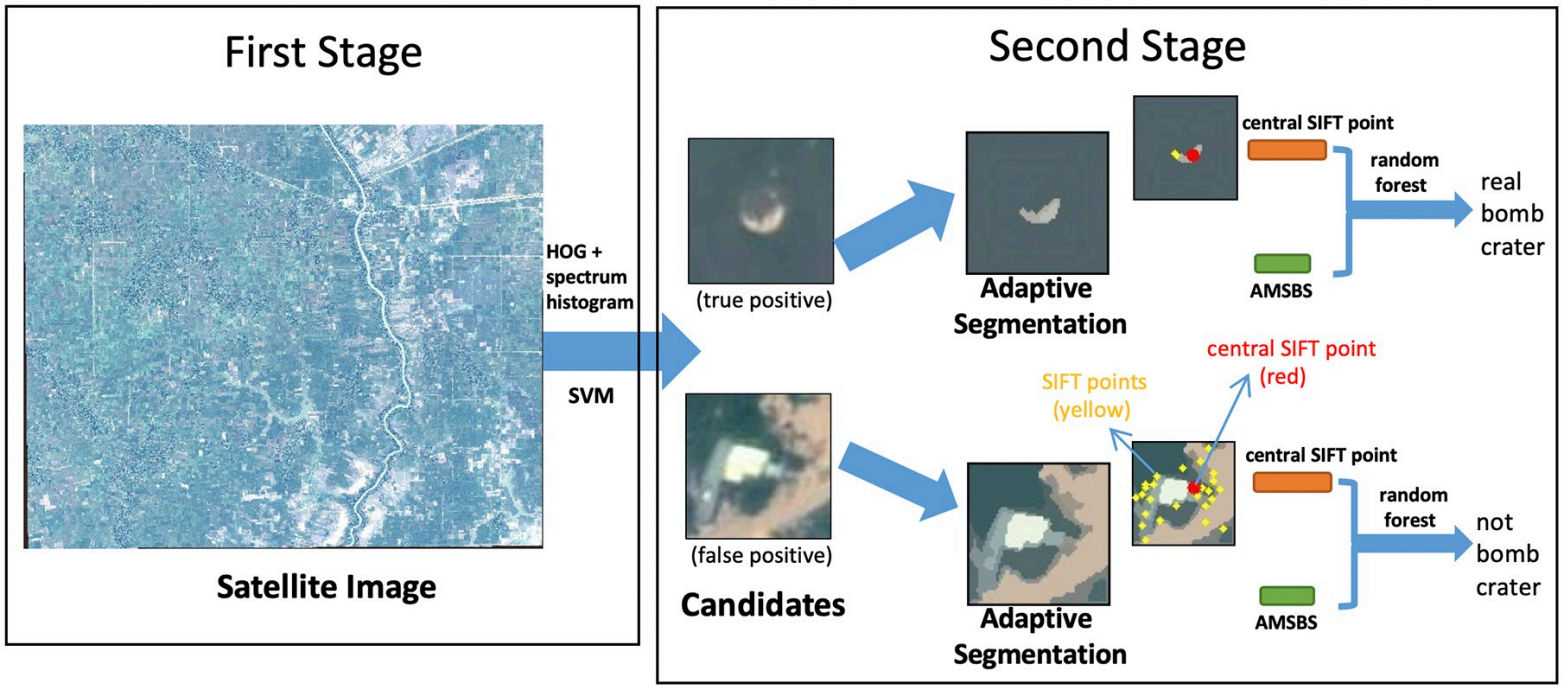
Workflow of the two-stage framework for bomb crater detection.

In the second stage, a novel method of feature extraction is built that first segments each candidate patch so that the circular object is separated from the surrounding region, a process that we call adaptive mean-shift segmentation. Then, the shape, location, and radiometric information is extracted out of the circular object, building a new adaptive mean-shift based shape (AMSBS) feature. Next, the textural patterns are extracted, using scale-invariant feature transform (SIFT) points. Finally, a random forest classifier is trained to use the AMSBS feature and the SIFT points in order to determine whether each patch candidate is a false positive or contains a real bomb crater. Fig 5 illustrates the workflow for our proposed method.

### Stage 1: Patch-based SVM classification using HOG and spectrum information

One of the defining features of a bomb crater is its circular shape. So for the first stage of processing, contour shape features are extracted using Histogram of Gradient (HOG), which is capable of describing objects with distinct contours in near circular shapes. It is robust to changes of illumination and shadow, and has been successfully applied to pedestrian detection in close range images [27].

However, the contour features of HOG may not be sufficient to distinguish craters, as there may exist other circular or near-circular objects, like ponds, silos, and cluster of trees or rocks. To address this issue, a histogram distribution of the spectral values of the patch is introduced to serve as another set of features to reflect the statistical spectrum of each patch, noted as *v*_*color*_ ∈ *R*^30^. Afterward, *v*_*color*_ is concatenated with the HOG feature. To account for noise in the feature vector, principal components analysis (PCA) transformation retains the first few components that preserve 0.9 of the cumulative sum of the eigenvalues. The obtained final feature vector of the first stage is noted as *v*_1_.

After training the SVM algorithm using the HOG + spectrum histogram feature, the classifier is tested on the satellite image by taking patches using a sliding window. The sliding window is a square that consists of 64 pixels (8 by 8), and scans in both horizontal and vertical directions. The SVM algorithm classifies each image crop inside the box, known as a patch, according to whether or not it contains the object of interest, circular shapes with coloring similar to the confirmed bomb craters. The classifier is able to separate almost all of the bomb craters from background terrains according to the experimental results. In other words, this first stage of processing is conservative enough to have retained most of the real bomb craters, but it comes at the expense of extracting many irrelevant objects with a similar appearance. The spectrum histogram identifies some incorrect patches along with some of the highly distinctive craters, so a large number of false positives are contained in the set of detected bomb craters. Our goal in the second stage is to develop a method that corrects the over-inclusion of false craters.

### Stage 2: Novel feature extraction and learning on random forest

In order to separate the false positives from the real bomb craters, a second stage of processing uses shape features more specifically designed for classifying bomb craters, such as area size and isotropy. Conventional classification approaches typically extract feature vectors using all the pixels of a patch. However, it is possible that one patch may contain other objects. For example, a patch with a bomb crater in its center region might have trees at the four corners, and these textures, if used in the feature vector, are likely to impact the detection results.

Compared to the human-identified craters, bomb craters present relatively homogeneous regions in terms of color. Given our selected detection method, a bomb crater is defined to be a circular object in the center of the patch. Therefore, the candidate patches are segmented to identify such patterns, using a mean-shift segmentation algorithm [31]. If a patch can be segmented to a few regions where the center regions are relatively isotropic and flat, there is chance that the patch may contain a bomb crater. However, if inappropriate parameters are used in the mean-shift segmentation, the patch may be over-segmented or under-segmented, leading to incorrectly identified patterns.

#### Adaptive mean-shift segmentation

Given the large variation of spectrum information across different image patches, no single parameter set will capture the range of bomb craters. Therefore, an adaptive mean-shift (MS) segmentation method is used to tune the associated parameters (i.e., the range bandwidth) of the classic MS algorithm [31].

**Fig 6:**
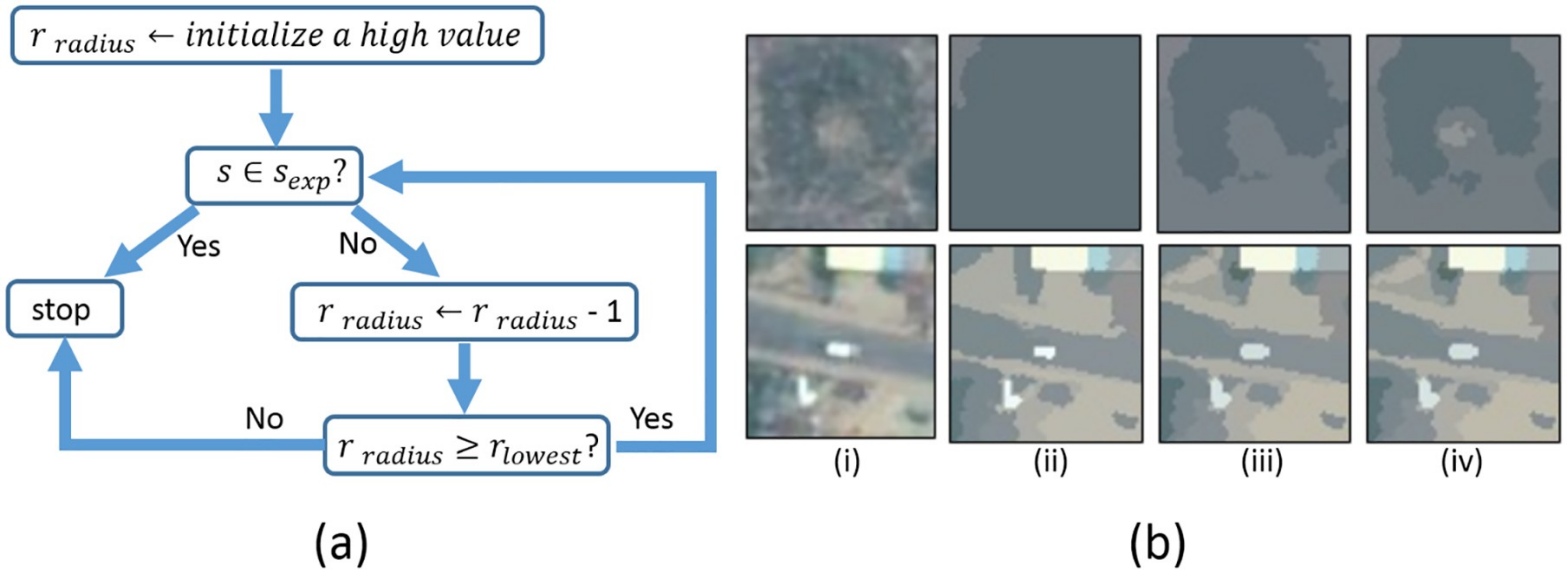
A diagram of the algorithm for our adaptive mean-shift (MS) segmentation (a) and an illustrative example of the adaptive MS segmentation process (b). An image of a real bomb crater is segmented in the top row, and an image of a building (a false positive) is segmented in the bottom row. Column i shows the original patches. Column ii, iii, and iv show the segmented results with a range radius of 5, 4, and 3 pixels respectively. The range bandwidth is reduced progressively until the central segment appears as specified.

Our adaptive MS method tunes the range radius *r*_*radius*_ dynamically and ensures that only one segment appears in the center of the patch. This central segment represents the object of interest (i.e., the bomb crater), where the features will be extracted. The range bandwidth parameter reflects the sensitivity of the algorithm when segmenting images; normally a large value refers to fewer large segments while a small value indicates more segments that are small in size. For each patch, we freeze the other parameters and initialize range bandwidth to a high value (5 in this case). Then the range bandwidth is monotonically decreased until the central segment appears with an expected segment, *e*_*exp*_. We define segment *s* to be contained within the expected segment (*s* ∈ *e*_*exp*_) when the most distant point in the segment is within 10 pixels from the center. If the central segment does not meet the size criterion, we discard the patch. This decision tree is illustrated in Fig 6a. Fig 6b traces the adaptive MS segmentation process through two example patches—a bomb crater and a building that the first-stage had identified as a bomb crater candidate.

#### Adaptive mean-shift based shape (AMSBS) feature

**Fig 7:**
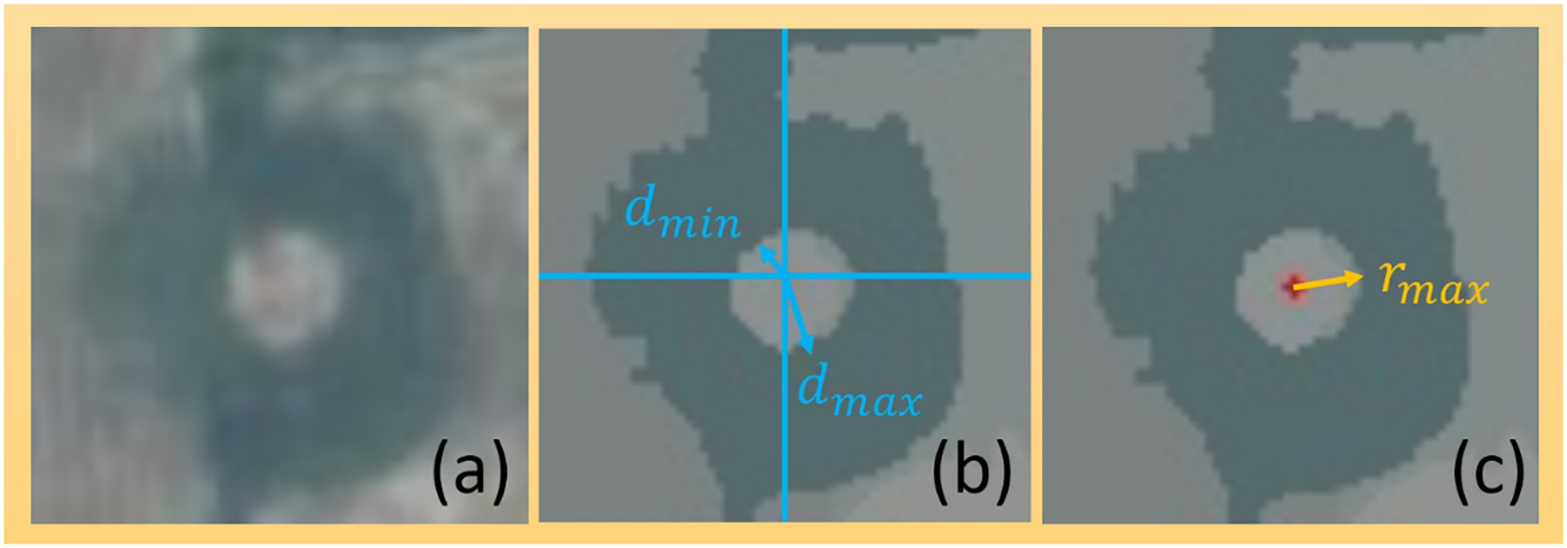
A visual example of the critical shape parameters that define the AMSBS feature. The original image of a crater (a) is measured to obtain the minimum and maximum distance from patch center to the segment boundaries, *d*_*min*_ and *d*_*max*_ (b), in addition to maximum compactness *r*_*max*_ (c).

Once the adaptive MS algorithm finds the specified segment in the center of the patch, the extracted features from this segment are used for classification. Since the features are extracted from regions adaptively defined by the segmentation algorithm, this shape feature is patch-dependent, and we call it the adaptive mean-shift based shape (AMSBS) feature. The AMSBS feature extracts the shape, location, and radiometric information out of the segment, and stacks the information as a feature vector. It is defined by (i) the centrality of the segment, or the maximum and minimum distance from the patch center to the segment’s boundaries, *d*_*max*_ and *d*_*min*_, as shown in Fig 7b and (ii) the maximum distance from the segment’s barycenter to the segment’s boundary, *r*_*max*_, as shown in Fig 7c. To simplify, hereafter we refer to it as the maximum radius of the segment. The algebraic description of the shape features is shown in Table 1. The shape features, stacked alongside the number of segments and the mean color values of the segment, constitute the AMSBS feature vector, as seen in Eq 1.

$$v_{0}=\left(\alpha ,d_{max},d_{min},r_{max},n_{groups},R,G,B\right)\in R^{8}$$ (1)

**Table 1:** Adaptive mean-shift based shape feature (AMSBS).

Description	Formula	Definitions
Occupancy ratio	$\alpha =\frac{s}{H\cdot W}$	*s* is the number of occupied pixels f the center segment. H and W are height and width of the patch.
Maximum distance to patch center	$d_{max}=max\left\{\sqrt{{\left(u-u_{center}\right)}^{2}+{\left(v-v_{center}\right)}^{2}}\right\}$	(*u*_*center*_, *v*_*center*_) is the center to patch center of the patch. (*u*, *v*) are points throughout the patch.
Minimum distance to patch center	$d_{min}=min\left\{\sqrt{{\left(u-u_{center}\right)}^{2}+{\left(v-v_{center}\right)}^{2}}\right\}$	
Maximum radius of center segment	$r_{max}=max\left\{1/2\sqrt{{\left(u-u_{center}\right)}^{2}+{\left(v-v_{center}\right)}^{2}}\right\}$	
Number of segments	*n*_*groups*_	Total number of segments in the patch. This serves as additional information about crater background
Spectrum	RGB	Spectrum of central segment

#### Central SIFT point

One additional feature vector is created to extract textural patterns from the central segment: the central SIFT point feature. The scale-invariant feature transform (SIFT) is a computer vision algorithm widely used in pattern recognition [33]. It extracts interest points over an image and forms a unique feature vector that describes the local textures.

In our framework, a 128-dimension SIFT feature vector extracts key point features from our human-coded bomb craters and our image patches. Since the target of concern is the central segment in the patch, only the feature vector associated with a detected point in the central segment is used. If more than one SIFT point is detected in the segment, then only the SIFT point closest to the patch center is used. This implementation does not lose any generality because the difference of descriptors among multiple SIFT points in the same segment is usually very small. See, for instance, Fig 5, in which the yellow dots indicate the detected SIFT points and the red dot represents the central SIFT point. To bring together all of the segment-specific information collected in the second stage, the central SIFT point feature vector *v*_*SIFT*_ ∈ *R*^128^ is concatenated with the AMSBS feature vector *v*_0_, estimating equations of the form:

$$v_{final}=\left(v_{0},v_{SIFT}\right)\in R^{136}$$ (2)

#### Binary classification using random forest

The objective of our second stage detection is to take the crater candidates from the first stage detection and refine the sample with more informative features. In our final step, a classifier is trained on the concatenated AMSBS feature and central SIFT point, *v*_*final*_. The scale distribution of each dimension of *v*_*final*_ lacks balance and varies significantly. With respect to categorical data, the random forest classifier has shown to be able to handle unbalanced distributions with reasonable accuracy [34]. The random forest is an ensemble classifier that uses a large number of decisions trees, providing an advantage over traditional classifiers [29]. Each tree is trained independently, and a mean predictor is taken over all trees. Consequently we use a random forest model with 850 decision trees. This classifier separates the crater candidates into two categories: likely bomb craters and false positives.

## Experiment and results

For the first stage of processing, the Scikit-image processing library extracts HOG feature with standard parameter sets [27, 35]. To calculate the spectrum histogram feature, the number of bins is set to 10 so that each bin covers a spectral bandwidth of 25.5. This ensures that most of the bins include some samples without making the histogram feature too sparse. Then principal components analysis (PCA) is applied to the concatenated HOG + spectrum histogram feature. The lowest decile of the transformed components is discarded to remove feature noise without losing dominant information [36]. Optimal parameters of SVM are identified by 10-fold cross validation using Scikit-Learn Library [36]; the penalty parameter *C* and kernel coefficient *γ* in the SVM are 0.0001 and 2.2 respectively. The sliding window stride is set to 8 pixels since this is large enough to accommodate the largest bomb crater, roughly 12 meters in diameter. It also saves computational cost compared to per-pixel sliding window approaches.

During the second stage of processing, the spatial bandwidth and minimal density in the means-shift segmentation were both set to 20 [31]. As mentioned earlier, the range bandwidth is initially assigned a relatively high value of 5 to achieve a relatively loose segmentation, and then is monotonically decreased by 1 in each iteration to get more refined segmentation until a central segment of the specified size appears. The segment must be from 25 to 624 pixels in size; given the WorldView 0.5 meter resolution image, this range covers all possible sizes of bomb craters, as indicated by the sample images provided by the international demining agencies. If a central segment of this size does not appear even when the range bandwidth has decreased to 1, the patch is coded as not containing a crater and discarded. When the SIFT points are detected, parameters are set to default using OpenCV throughout the second stage [37]. This specification helps avoid missing important feature points in the segmented texture-less patch.

**Fig 8:**
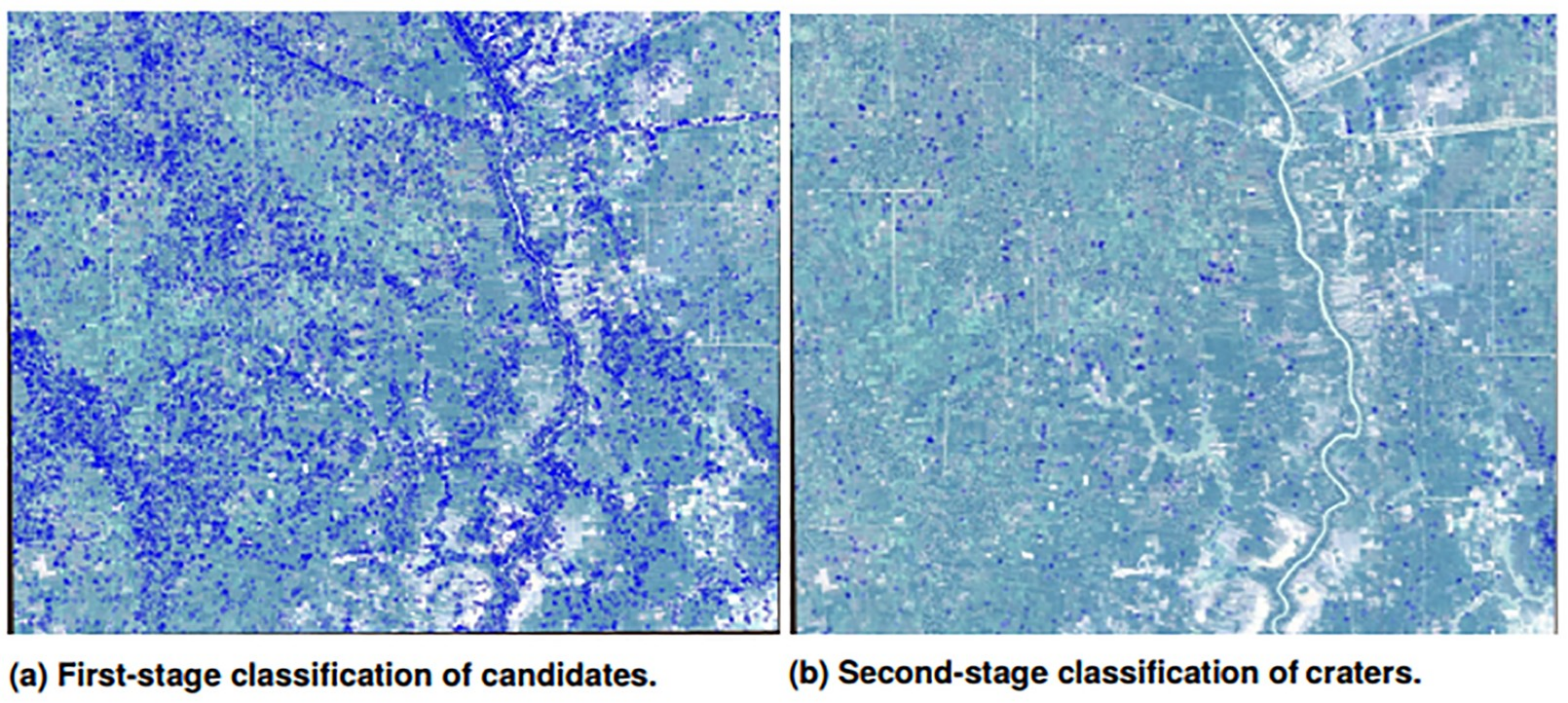
Detection results over the entire WorldView image. Eighty-three percent of the crater candidates from the first stage were dropped after the second stage refinement.

We apply our two-stage detection framework to the entire WorldView image, and provide the classification results of each stage in Fig 8. On the left side, the crater candidates detected after the first stage are highlighted in blue. There are 22,366 candidate patches detected on the entire image. On the right side, the 1,585 craters identified as likely bomb craters after the second stage. Roughly 83% of the crater candidates were discarded as false positives.

In order to evaluate the accuracy of our new method, we compare our two-stage framework to two alternatives, HOG + SVM and Convolutional Neural Network, for accuracy comparison purposes. All the bomb craters were manually extracted for validation on a small test region. The human coder identified 177 bomb craters on the validation region. Our method finds 1299 bomb craters after the first stage; 157 are bomb craters while the other 1142 are false positives. Therefore, the first stage is able to find 89% of the bomb craters but it also finds many false positives. After the second stage refinement, the 1299 detection candidates are reduced to 207. Among these, 152 are bomb craters and the other 55 are false positives, so the two-stage framework has an accuracy of 85.9% (152/177). The second stage successfully eliminates 96% of the false positives while it also preserves the number of real bomb craters, only losing five.

We also conduct comparative experiments to demonstrate the effectiveness of our approach. Since no other algorithms that we are aware of address crater detection on natural terrains, we compare our framework with state-of-the-art object-recognition methods, HOG+SVM [25] and Convolutional Neural Network (CNN) [38–40]. In order to elicit a fair comparison, the standard HOG+SVM approach and CNN extracted feature with SVM (CNN+SVM) approach are applied to the satellite image in the same sliding window manner as our first stage framework. The HOG feature parameter settings are identified by 10-fold cross validation with grid search, as was done in the first stage of framework. We also adopt the state-of-the-art CNN architecture, VGG-16, as the basic CNN feature extractor [41]. Its parameters, such as kernel weights and bias, are loaded from previously trained values on ImageNet [40]. The fully connected layers used for classification are removed, and only the CNN and pooling layers in front are kept for feature extraction. Patches are warped from 64 × 64 to 224 × 224 before feeding them to VGG-16. The output dimension from the neural network is 7 × 7 × 512, which is then reshaped into a vector with a total dimension of 25,088. After applying PCA, the feature vector dimension is reduced to 379 to keep the dominant feature information [42].

Then, the second stage of our framework is compared to Bag of Words (BoW) and CNN feature maps, which are both able to detect false positives [43]. These comparative experiments are performed on the candidate patches from the first stage of our method. They also both use a random forest classifier, like our method. The parameters are defined accordingly: number of trees *n*_*tree*_ and the maximum depth of the tree *depth*_*max*_ are identified by 10-fold cross validation with grid search. To determine the SIFT points, the BoW features were built using K-means clustering with the SIFT points located in the central segment [44]. The number of clusters is set to 15 because it is the smallest value that can sufficiently distinguish SIFT feature samples. We have observed in other repetitive tests that other possible parameter settings are able to achieve a similar performance. For CNN features, VGG-16 is used again as the basic CNN feature extractor on the segmented patch. The same pre-processing steps are used as in the first stage comparison. Then, PCA is applied to the features extracted from CNN to reduce dimensions before classification.

Our evaluation of model performance is based on three metrics commonly used in machine learning: F1-score, recall and precision [45]. Their definitions follow:

$$F_{1}=\frac{2\cdot \text{recall}\cdot precision}{precision+recall}$$ (3)

$$recall=\frac{number\;of\;bomb\;craters\;detected}{number\;of\;total\;bomb\;craters}$$ (4)

$$precision=\frac{number\;of\;bomb\;craters\;detected}{number\;of\;detection\;patches}$$ (5)

**Fig 9:**
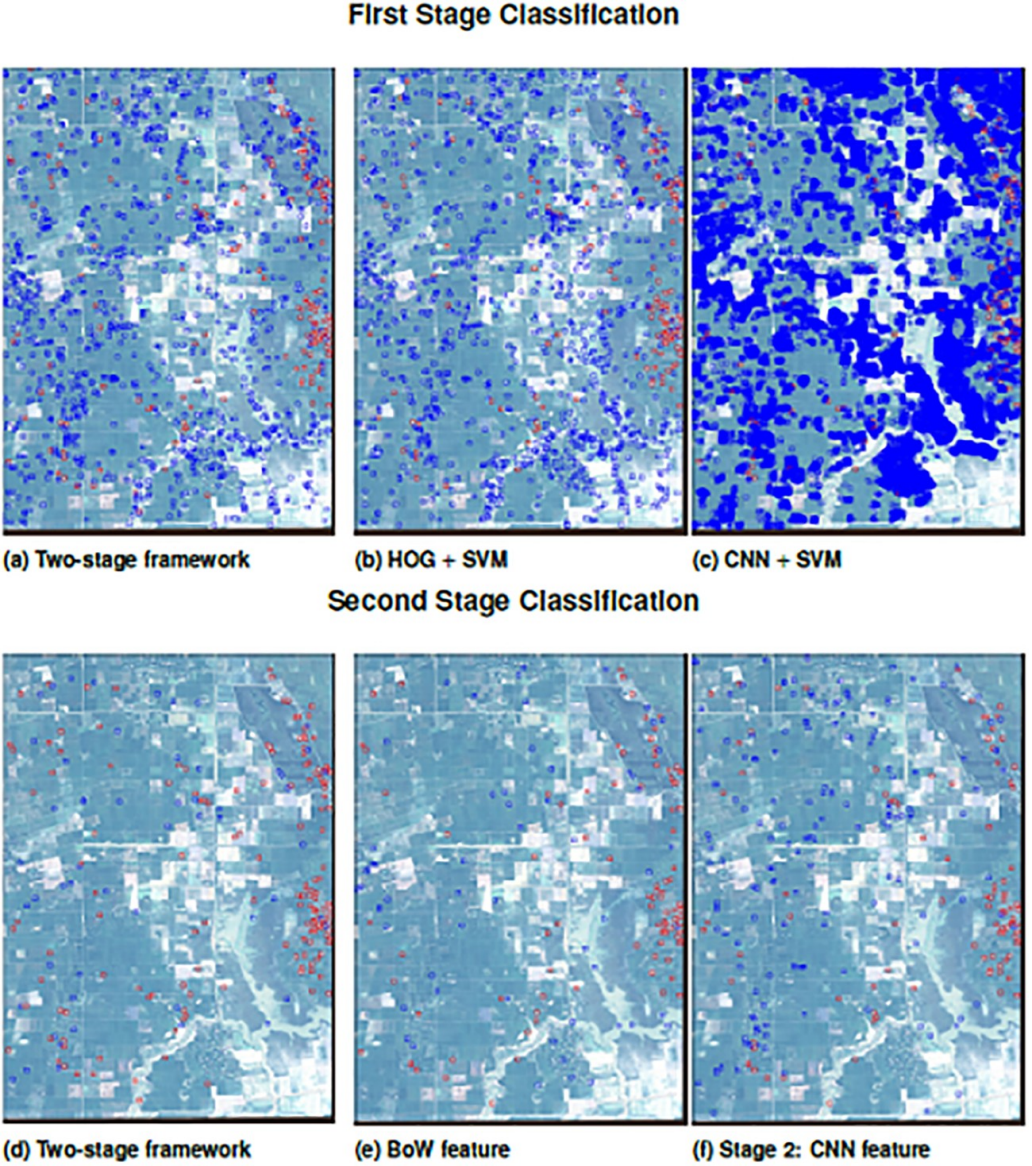
Results of candidate detection (first stage) and crater refinement (second stage), comparing our two-stage framework to widely-used alternatives. A red box indicates the model correctly found a bomb crater. A blue box indicates the model found a false positive.

Statistical results are presented in Table 2, and patch detection is visualized across the two stages in Fig 9. Our method’s first stage has a high recall value (0.89), ensuring that crater candidates detected in this stage include most of the actual bomb craters, which will be important for the second stage refinement. Our first stage alone out-performs the traditional HOG+ SVM and CNN+ SVM approaches in each metric, though the discrepancy is not large and the alternative methods are still comparable to ours.

**Table 2:** Accuracy assessment our two-stage framework and two alternative approaches.

	*Model Performance*		
	Precision	Recall	F1-Score
*Candidate Detection (first stage)*			
Our First Stage	0.121	0.887	0.213
HOG+SVM	0.010	0.763	0.176
CNN+SVM	0.006	0.740	0.011
*Crater Refinement (second stage)*			
Our Second Stage	0.734	0.859	0.792
BoW Feature	0.612	0.525	0.565
CNN Feature	0.364	0.531	0.432

In addition, our second-stage features are able to further refine the detection results with higher precision. Note that our method’s second-stage result is equivalent to the final result of this whole two stage framework. Our proposed feature representation of AMSBS plus central SIFT obtains the highest score for each metric, outperforming the other classification options. Notably, our two-stage framework has an F1-score of 0.79 compared to 0.57 of BoW and 0.43 of CNN feature. Additionally, the precision level after the second stage has tremendously increased from 0.12 to 0.73, with only a slight drop in recall value (from 0.89 to 0.86). The proposed method increased true bomb crater detection by over 160 percent. Our second-stage processing effectively removes a large portion of the false detection without dropping the real bomb craters, which illustrates the main advantage of this two-stage framework.

In short, when compared to alternative methods, our two-stage framework reports improved results and can be easily applied with a limited number of training samples, requiring minimal human labor involvement. Our proposed framework can also be modified so that if new method outperforms either of the two stages, it can be integrated into our proposed workflow.

## Application of results to UXO estimation and post-conflict reconstruction

The results from our two-stage framework can help demining organizations proactively locate areas that have a high density of unexploded ordnance. Since a bomb crater provides physical evidence of a successful detonation, we are able to estimate the number of bombs that have not detonated within the target buffer, thereby providing more detailed locations of areas that need professional clearance. A single B-52 payload holds up to 108 225-kilogram or 42 340-kilogram bombs, which were dropped on a target area of 500 by 1500 meters [46]. The declassified US Air Force dataset indicates that 3,205 general purpose bombs, more commonly known as “carpet bombs,” were dropped over the 100 km^2^ area represented in the satellite image. Following a recommendation from an international demining agency working in Cambodia, we draw slightly larger buffers around each payload coordinates (1,750 meters in diameter) to compensate for the human error in reporting the coordinates.

**Fig 10:**
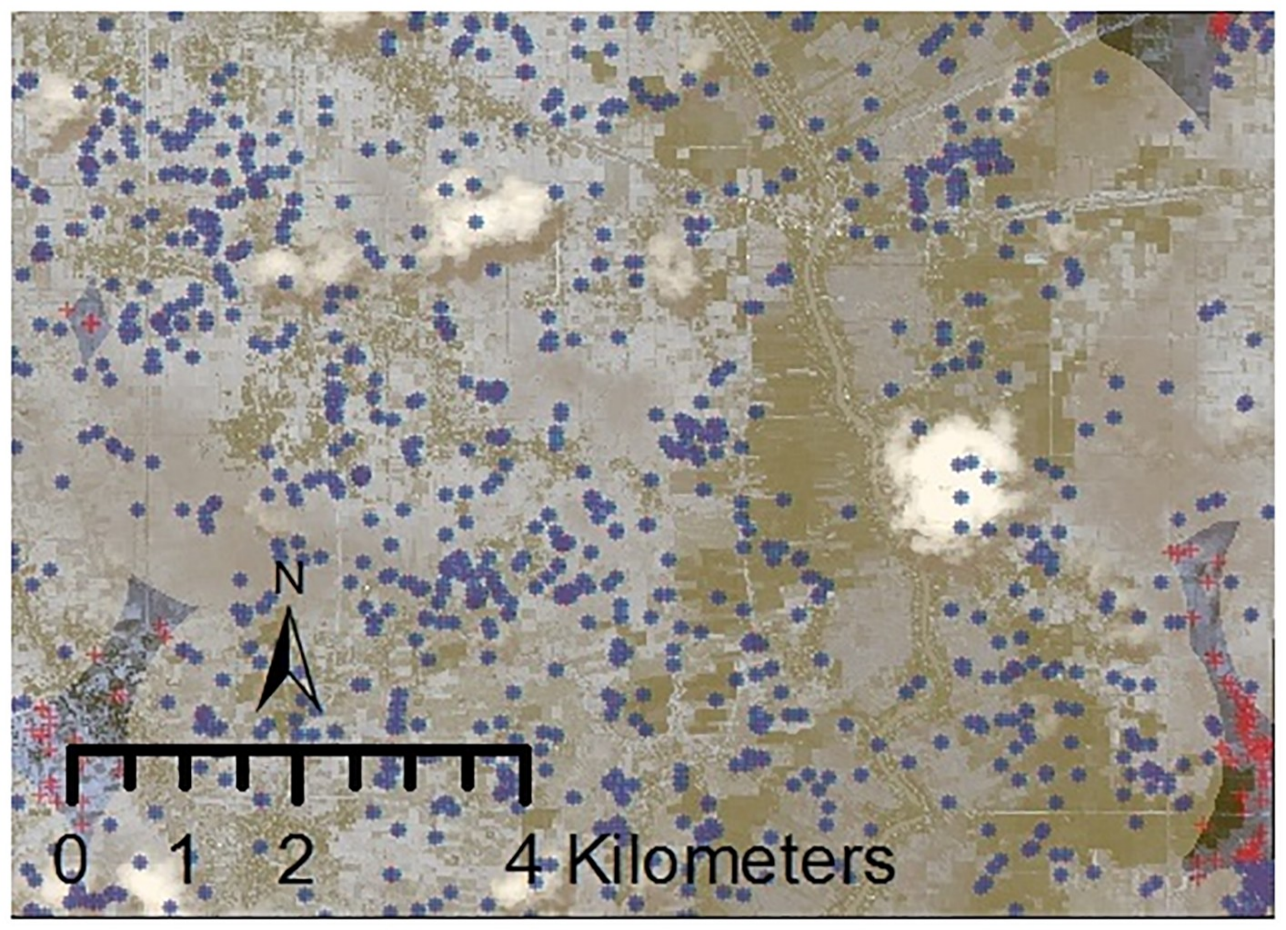
Payload drop zone for the US airstrikes over the satellite image during the Vietnam War. Over 98 percent of detected craters fall within 1,750 meters from a payload drop coordinates. These craters are highlighted in blue while craters outside the target buffers are represented in red.

First, an accuracy assessment is performed by triangulating our model’s detection results with ground reference information. Since information about each UXO location is limited, we rely on the US Air Force dataset combined with the experience of the international demining agencies to draw “most-likely” spaces of where we expect to find bombs—both detonated (craters) and undetonated (UXO). Fig 10, we draw the effective target zones—that is, the buffers surrounding each payload drop—to illustrate how our model finds craters where we would expect to see them. Almost all of the craters detected by our model (98%) are found within these buffers, i.e., within 1,750 meters of a payload drop coordinates, suggesting that our model performs high degree of accuracy.

Then, our model’s performance statistics are used to estimate the number of bomb craters on the image, compared to the number of craters detected by our model. When our model is applied over the entire satellite image, it detects 1,585 craters. Given our two-stage framework’s recall value of 0.86, there could be an estimated 1,843 total craters on the overall image. At a minimum, our model detects 1,585 craters while our best estimate is 1,843 craters. When the estimated number of craters is subtracted from the total number of bombs dropped in this area, we estimate that 1,407 to 1,620 bombs are undetonated. While a professional demining agency had cleared a small field within this area, they had only found two general purpose bombs. As discussed above, the cleared field reflects 0.12% of the entire image; incidentally two bombs represent 0.12% of an estimated 1,620 undetonated bombs, providing some indication that our predictions reflect real-world UXO density. In sum, anywhere from 1,405 to 1,618 unexploded carpet bombs are still unaccounted for in this area. Combined with declassified US Air Force records and demining reports, our results suggests that 44 to 50 percent of carpet bombs remain unexploded around this particular Cambodian village.

**Fig 11:**
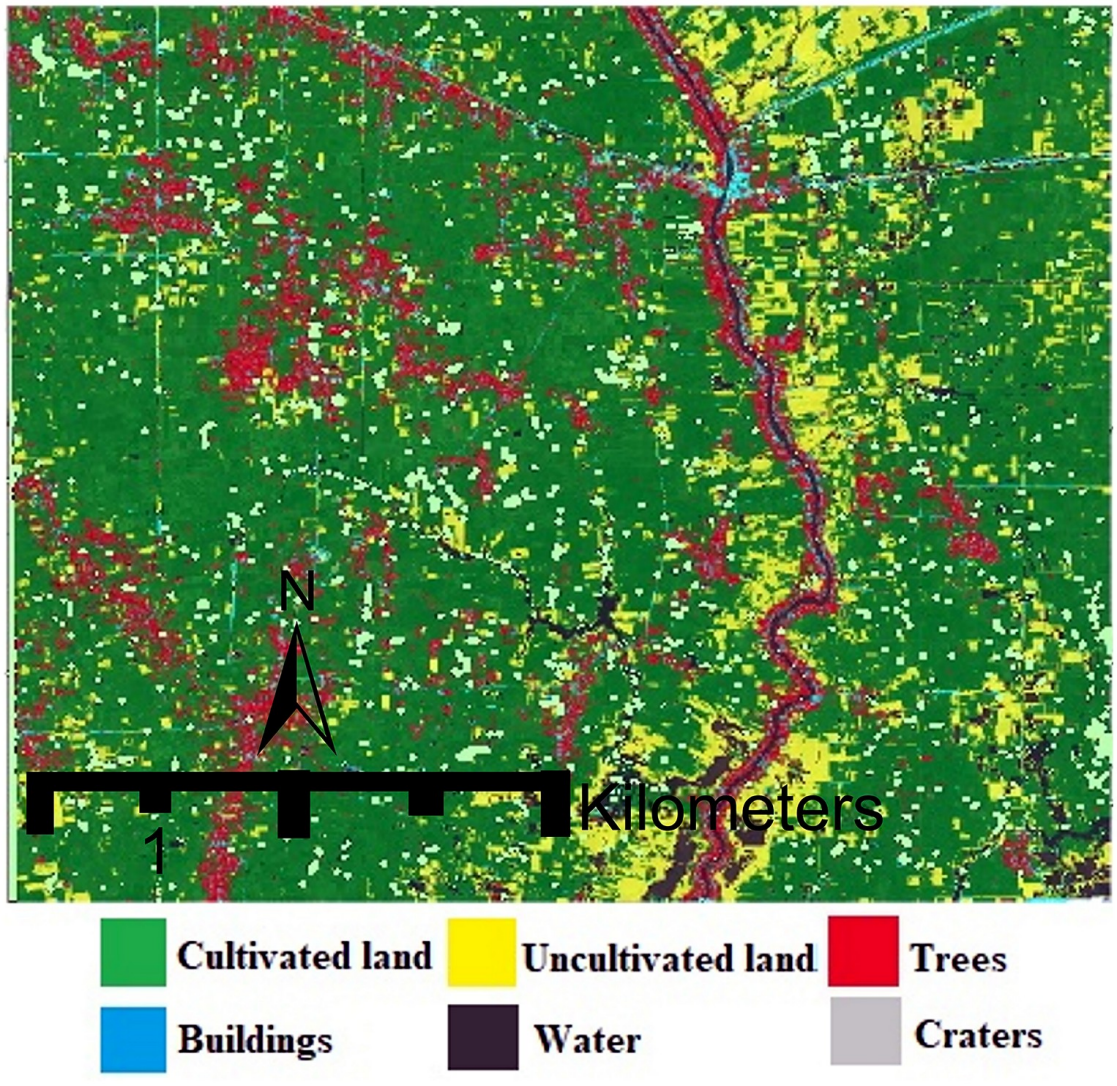
Land classification for the satellite image. Land cover classification suggests that the majority of the land surrounding Kampong Trabaek is actively cultivated, despite likely UXO contamination. The gray squares represent detected craters.

**Fig 12:**
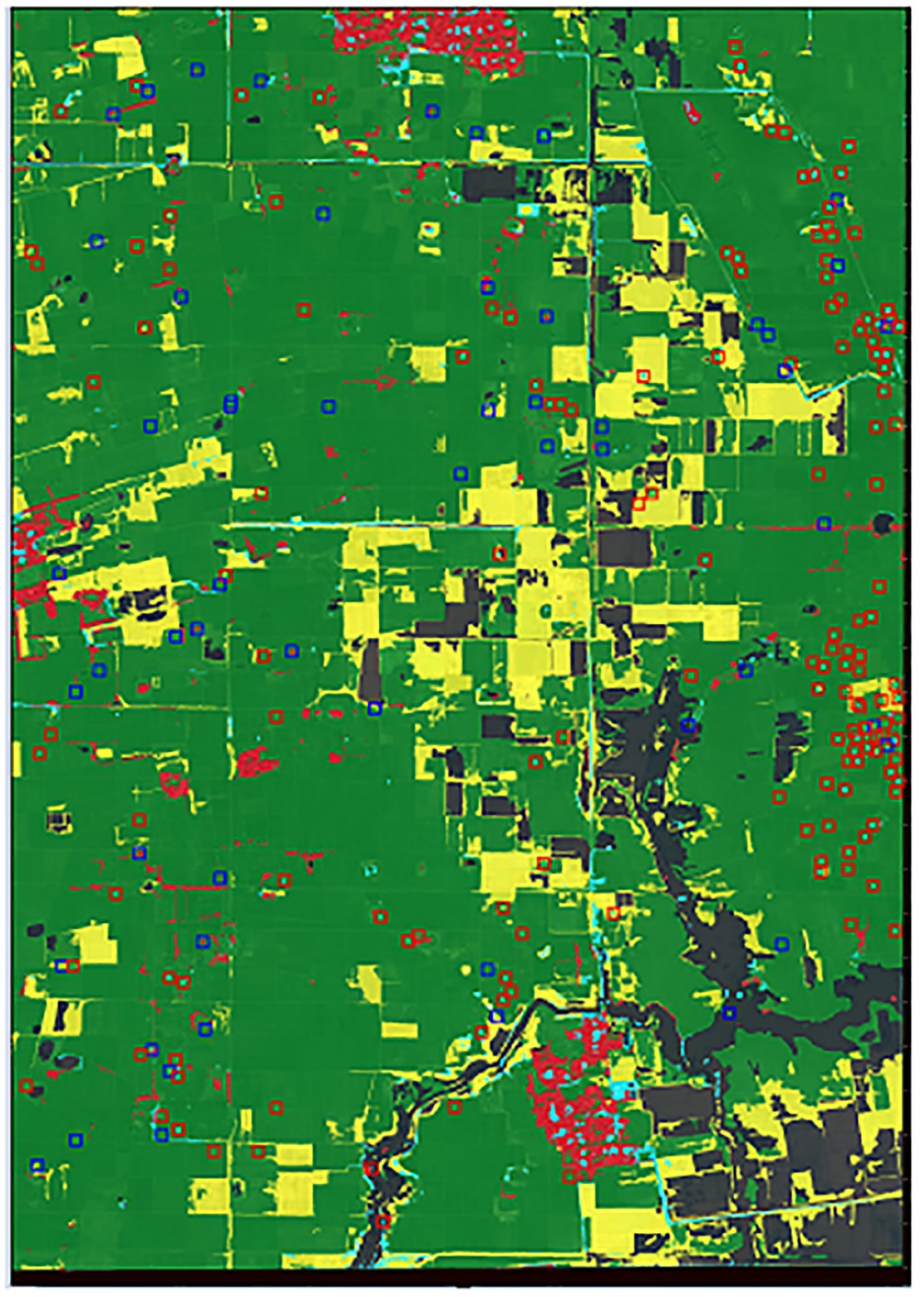
Land classification within the validation region. A close-up of the validation region shows that the two-stage framework has reliable accuracy across cultivated, uncultivated, and developed land. The red squares are false positives and the blue squares are true positives.

Although our results suggest that a substantial number of unexploded bombs are likely to be left within this 100 km^2^ region, demining agencies may not want to devote scarce resources to clearing areas that are not accessible or widely used. Therefore, we provide land cover classifications for the VHR image to assess how the contaminated land surrounding Kampong Trabaek village is being used. The land classification data are generated using eCognition software and the object-based classification method, which divides the image into six classes: cultivated agricultural land, uncultivated land, buildings, water, trees, and clouds. The results show that the majority of the experimental satellite image is cultivated agricultural land (see Fig 11) while a close-up of the validation region describes our two-stage model’s accuracy across land classes (see Fig 12).

Across varying terrain, the two-stage framework has high precision and recall (see Table 3). The density statistics indicate that bomb craters are found likely to be found in across all land classes. This pattern reflects the indiscriminate nature of the carpet bombing, in which bombers dropped payloads at such high altitudes that they had near zero visibility of targets on the ground. These conditions made the damages of the air raids widespread across all types of land. The model’s high detection rate of craters in tree-covered areas indicates the similarity between the shapes and textures of tree groves and craters, and motivates further inquiry for future models.

**Table 3:** The two-stage framework results from *validation region* indicates a nearly equal density of craters on cultivated, uncultivated, and forested land. There tends to be a higher density of craters near residential buildings, suggesting that households are actively living in bombed areas.

	Crater Count			Density (Count/*km*^2^)		
	Total	Detected	True Positive	Total	Detected	True Positive
Cultivated	143	147	121	23.3	24.0	20.8
Uncultivated	23	30	18	23.2	30.3	18.2
Tree cover	4	18	4	20.8	93.8	20.8
Residential building	5	6	4	51.1	61.3	40.9

## Discussion

Our technical contribution includes a two-stage framework that integrates segmentation [47] and detection [48] as key tasks for crater detection. Through extensive experiments and comparative analysis, we demonstrate that our method significantly outperforms typical object detection algorithms. Moreover, the proposed two-stage framework requires only a limited amount of data for learning, i.e. 157 labeled samples. The effectiveness and data-efficiency of this two-stage framework can dramatically alleviate human labeling labor. This two stage framework is also easily modifiable and amendable such that any method outperforms in any of the two stages, and can be integrated into our proposed workflow to achieve satisfactory results. We hope this framework can provide ideas for similar wide-area bomb crater detection tasks.

The presented study shows that Very High Resolution satellite images not only deliver sound information on bomb crater density, but also provide detailed insight into UXO exposure and the complex surface dynamics related to small-scale agricultural activities. In particular, a combination of a HOG and spectral information classifier and a novel patch-dependent spatial feature that adapts to different crater sizes and terrains reveals that 44 to 50 percent of carpet bombs are still unaccounted for. Nevertheless, crop cultivation goes on, documented by the actively cultivated land surrounding the craters and payload drops. This observation matches well with reports summarizing that Cambodian farmers adapt to these dangerous living conditions by changing their land management practices [49, 50]. The current study and the results of the detailed analysis further complement the scholarly findings, providing explicit spatial information on the extent to which contaminated areas are still farmed. It suggests that future research can use novel land classification techniques to quantify the agricultural productivity on UXO-contaminated land for further comparison with safe land.

Still, identifying a bomb crater does not provide definitive proof of a detonated bomb, but serves more as an indication. By triangulating our model’s findings with declassified US Air Force records and deminer interviews, we can substantiate our assessment while we also acknowledge the role that future research can play in verifying these findings with, for example, farmer surveys and soil tests that can confirm the existence of explosive material within the crater candidate. Although crater verification is a standard issue for all remote sensing methods, one of the advantages of remote sensing is that it can be applied in more remote and insecure areas, where deminers may be unsure if they should spend precious resources on a scoping mission. A two-stage approach, such as the one described here, can detect bomb craters more efficiently than alternative, out-of-the-box approaches.

## Conclusion

The identification and removal of UXO have been recognized as key to long-term economic development and peace-building in post-conflict countries [4, 51]. In the six decades following the secret bombing of Cambodia, over 64,000 people have been killed or injured by UXO, and today the injury count averages one person every week. In Afghanistan, UXO from the post 9/11 airstrikes, which relied on carpet bombing and dropping cluster munitions, restricted farmers’ access to fields and shepherds’ access to pastures, as well as other disrupting daily routines to schools, markets, and neighboring villages [52, 53]. The presence of UXO and mines in Pakistan has encouraged many Kashmir residents to move to refugee camps, due to loss of jobs and poor access to agricultural lands [54]. Even where weapons testing took place in Vieques, Puerto Rico, dangerously high levels of carcinogens were found in the waters and coral reefs surrounding the corroding live bombs dropped by the US Navy [55]. It is alleged to contribute to unusually high rates of cancer in fish-consuming households near the exposed reef [56]. Many of the most dangerous areas in Syria, Afghanistan, Libya, Ukraine, and Sudan are littered with unexploded ordnance dropped by international or rebel forces. In these post-conflict settings, scores of stabilization, development, and peacekeeping missions are taking place in literal minefields, where we have little information on hot spot boundaries and the location of explosive remnants of war.

A remote-sensing method that identifies the location of UXO has many downstream applications, such as helping operational teams more safely traverse conflict-affected regions. Beyond logistical support, this method can also help guide policy to set the foundations for long-term growth in areas that still suffer from the threat of violence. For instance, since the process of demining is an expensive and time-intensive one, this framework helps identify the most vulnerable areas that should be demined first. Meanwhile, more studies are needed to inform how, in post-conflict regions, extra food and economic aid may need to be distributed to UXO-dense areas and which local health clinics and social services may need to prepare for UXO explosions.
